# Ambient air pollution and meteorological factors associated with hand, foot, and mouth disease risk in Shenzhen, China: a time-series study

**DOI:** 10.3389/fpubh.2026.1920424

**Published:** 2026-10-06

**Authors:** Shuai Jiang, Jingwen Liu, Jiajia Ji, Yanpeng Cheng, Zhen Zhang, Guomin Chen, Zhigao Chen, Xiaoheng Li, Huawei Xiong

**Affiliations:** 1 Shenzhen Center for Disease Control and Prevention, Shenzhen, China; 2 Faculty of Health and Medical Sciences, The University of Adelaide, Adelaide, SA, Australia

**Keywords:** air pollution, DLNM, effect, HFMD, meteorological variables

## Abstract

**Background:**

In China, hand, foot, and mouth disease (HFMD) persists as a prevalent infectious disease with high transmissibility. Nevertheless, the link between environmental determinants and HFMD incidence has not been fully elucidated. The study assessed associations between air pollution, meteorological conditions, and HFMD incidence in Shenzhen, China, while also evaluating shifts in these associations before and after the Enterovirus 71 vaccine and during the COVID-19 epidemic.

**Methods:**

Daily data on HFMD cases, air pollutants (such as PM_2.5_, PM_10_, SO_2_, NO_2_, CO, and O_3_), and meteorological variables (including mean temperature, relative humidity, atmospheric pressure, and wind speed) were collected for the period from 2013 to 2022. Associations were assessed using distributed lag non-linear models (DLNM), accounting for immediate and delayed effects.

**Results:**

Spearman correlation analysis showed that HFMD was positively correlated with SO₂, mean temperature, relative humidity, and wind speed, and negatively with PM₂.₅, PM₁₀, NO₂, CO, and atmospheric pressure. DLNM models revealed that SO₂ and CO both elevated cumulative risk at extremely high concentrations, with SO₂ showing delayed effects. However, SO₂ unexpectedly displayed protective effects in single-lag analysis. NO₂ exhibited a U-shaped risk at extreme concentrations despite an overall decreasing trend. O₃ showed dual peaks, with protective effects at extremely high concentrations after lag 1. PM_2.5_ and PM_10_ showed risk peaks at low concentrations but protective effects at extremely high concentrations. Temperature exhibited a dual-peak risk pattern at extremely low and extremely high temperatures, with extreme heat producing sustained effects and extreme cold shorter-lived risk. HFMD risk increased under conditions of extremely high humidity. In general, the risk associated with atmospheric pressure rose as pressure levels increased, and asymmetric lagged effects were observed at both low- and high-concentration extremes. Extremely high wind speed causing short-term harm and extremely low wind speed leading to delayed cumulative risk.

**Conclusion:**

Meteorological factors exhibit more consistent associations than air pollutants. Mean temperature showed a dual-peak distribution with persistent effects from high temperatures, while atmospheric pressure presented long-lasting impacts of low pressure. There is a positive correlation between relative humidity and the incidence of HFMD. Air pollutants showed inconsistent associations with HFMD. These findings support targeted public health interventions for meteorological factors.

## Background

Hand, foot, and mouth disease (HFMD) constitutes a prevalent viral infection, mainly impacting children aged less than 5 years (1–3). While HFMD is generally mild and resolves within a week, it presents with fever, vesicular rashes on the extremities, and ulcerative lesions in the oral cavity due to blister rupture (4, 5). However, some patients may develop severe complications, including neurological or cardiopulmonary issues, which can be fatal. Human enterovirus A71 (EV71) and coxsackieviruses A10 and A16 serve as the primary causative agents of HFMD, with EV71 frequently linked to more severe outcomes (6)—including neurological sequela (7). EV71, a positive-sense RNA virus of species A (Enterovirus genus, Picornaviridae family) (8, 9), has been identified as a leading etiological agent in severe and fatal HFMD infections. The disease imposes a substantial burden in the Asia-Pacific region, where large recurrent epidemics have been documented since the late 1990s (10). The disease is primarily transmitted through three main routes: person-to-person contact, inhalation of respiratory droplets, and the fecal–oral pathway (11).

Air contamination has been more widely recognized as a key environmental risk factor that promotes the onset of HFMD. Exposure to air contaminants—including PM_2.5_, PM_10_, NO_2_, and O_3_—has been shown to correlate with a higher occurrence of HFMD (12, 13). Exposure to air pollution may enhance susceptibility to HFMD by compromising respiratory function in children (14) and increasing the environmental persistence of the virus (15). Studies have also shown that meteorological factors, including temperature, humidity, air pressure, and wind speed (12, 16, 17), can affect viral transmission and lead to a rise in the number of HFMD cases (15).

Over the past decade, Shenzhen has continued to report a high burden of HFMD, totalling 473,695 cases from 2013 to 2022, with an average annual incidence far exceeding the national level (10). Over the same period, Shenzhen has experienced significant air quality changes with trends of decreasing PM_2.5_ concentrations alongside rising ground-level O_3_ (18). Meanwhile, the city’s subtropical monsoon climate, rapid urbanization, and rising heat load further complicate disease dynamics (19). Since August 2016, the city has promoted EV71 vaccination among eligible children and wide EV71 vaccination coverage has contributed to declining incidence and severe cases of EV71-associated HFMD (20). In addition, strict COVID-19 prevention and control measures were implemented from January 2020 through the end of 2022, which exerted a notable impact on the incidence of HFMD.

Although previous studies have examined HFMD in Shenzhen (21–23), most were conducted before the recent shifts in environmental factors, pathogen composition or infectious disease campaigns. The dynamic changes over recent years require an updated investigation. This study addresses these gaps by integrating recent surveillance, air quality, and meteorological data, and assessing the effects of both the EV71 vaccine rollout and the COVID-19 pandemic. Enhanced understanding of these factors will not only aid relevant departments in detecting and responding to potential HFMD outbreaks in the future, but also provide evidence-based references for optimizing environmental health management, refining urban planning that aligns with public health needs, and formulating more infectious disease prevention and control policies.

## Methods

### Research area description

Shenzhen, situated in the southern part of Guangdong Province, is a coastal metropolis sharing its border with Hong Kong and Macao. The city extends over 1,997 square kilometers and falls within the subtropical monsoon climate zone, experiencing an average annual temperature of nearly 23 °C. Over the 2013–2022 study period, Shenzhen’s annual permanent resident population stood between 12.57 million and 17.66 million, recording an average population of around 15.75 million.^1^ Geographically, Shenzhen lies within the coordinates 113.43°–114.38° E and 22.24°–22.52° N.^2^

### Data collection

Daily reported cases of HFMD covering the period 2013 to 2022 were collected from the infectious disease surveillance networks of Shenzhen Center for Disease Control and Prevention. Daily data spanning the same period, which included daily concentrations of air pollutants (PM_2.5_, PM_10_, SO_2_, NO_2_, CO, and O_3_) as well as meteorological conditions (mean temperature, relative humidity, atmospheric pressure, and wind speed), were acquired from Shenzhen Ecological Environment Monitoring Center Station and Meteorological Bureau of Shenzhen Municipality.^3^

### Statistical analyses

Descriptive statistics summarized HFMD and environmental variables. To evaluate the associations between HFMD incidence and air pollution as well as meteorological factors, Spearman correlation analysis was conducted. The Spearman correlation coefficient was calculated as:

$$r_{s}=1-\frac{6\sum_{i=1}^{n}d_{i}^{2}}{n(n^{2}-1)}$$

Where *d_i_* represents the difference between the ranks of paired observations for the i-th pair, and n is the number of observations. The Spearman coefficient ranges from −1 to +1, with positive values indicating a positive monotonic association and negative values indicating an inverse association. Statistical significance was defined as a two-sided *p*-value < 0.05.

In line with earlier research, a quasi-Poisson regression framework incorporating distributed lag nonlinear models (DLNM) was used to examine the associations between air pollutants and meteorological conditions on HFMD occurrence, taking into account data distribution characteristics and inter-variable relationships. The model is specified as follows:

$$\begin{array}{l} \log[E(\mathrm{Yt})]=\alpha +\mathrm{cb}(X,\mathrm{df}=3,\mathrm{lag}=21)+\mathrm{ns}(\text{Time},\mathrm{df}=6/\text{year})\\ +\mathrm{\Sigma ns}(\mathrm{Xi},\mathrm{df}=3)+\text{βDOWt}+\text{γHOLIDAYt}+\text{δVACt}+\text{εCOVt} \end{array}$$

Where α represents the intercept, and Yt is the number of HFMD cases observed on day t. The term cb(·) denotes the cross-basis function constructed with 3 df for the exposure dimension (natural cubic splines with knots placed at the 33rd and 67th percentiles) and 3 df for the lag dimension (natural cubic splines), with a maximum lag of 21 days. Each of the 10 environmental variables — PM_2.5_, PM_10_, SO_2_, NO_2_, CO, O_3_, mean temperature, relative humidity, atmospheric pressure, and wind speed — was separately examined as the primary exposure.

For each model, natural cubic spline functions ns(·) with 3 df were applied to the remaining environmental variables (Xi) to adjust for their potential confounding effects. Given the high collinearity between PM_2.5_ and PM_10_ [Spearman ρ > 0.85; variance inflation factor (VIF) > 5; Supplementary Table S4], these two particulate matter fractions were not included simultaneously in any model. Specifically, when a meteorological variable (temperature, relative humidity, atmospheric pressure, or wind speed) served as the primary exposure, the covariates comprised the remaining meteorological variables together with CO, SO_2_, NO_2_, O_3_, and PM_2.5_ (PM_10_ excluded). When CO, SO_2_, NO_2_, or O_3_ served as the primary exposure, the covariates comprised the other three gaseous pollutants, all four meteorological variables, and PM_2.5_ (PM_10_ excluded). When PM_2.5_ or PM_10_ served as the primary exposure, the covariates comprised all four meteorological variables and the four gaseous pollutants (CO, SO_2_, NO_2_, O_3_), with the alternate particulate matter fraction excluded.

The Time variable was specified with 6 df per year to capture long-term temporal trends and seasonal patterns, selected based on the quasi-Akaike information criterion (QAIC) (Supplementary Table S1). DOW and HOLIDAY denote the effects of the day of the week and public holidays. VAC and COV indicate whether the date fell within the EV71 vaccination or COVID-19 prevention period. The maximum lag of 21 days was selected based on QAIC comparison across 7, 14, and 21 days (Supplementary Table S2), and is consistent with the incubation period of enteroviruses and previous HFMD time-series studies (24). Knot placement at the 33rd and 67th percentiles was selected based on QAIC comparison with alternative strategies (Supplementary Table S3). As a sensitivity analysis to evaluate the potential impact of the COVID-19 pandemic, we repeated analyses excluding the pandemic period (2020–2022). Cumulative RRs at the 95th percentile were compared between the full-period (2013–2022) and pre-pandemic (2013–2019) datasets (Supplementary Table S5). We considered an association stable if both its direction and statistical significance remained consistent across the two datasets; associations that changed in direction or significance upon exclusion of the pandemic period were deemed sensitive to the inclusion of the pandemic period and are interpreted with caution throughout.

Cumulative effects of environmental exposure variables on HFMD risk were measured for lags 0–21 days, using the meadian (50th) of each variable as a reference (Table 1). The effects of extreme exposures were expressed as relative risks (RRs), comparing the 5th and 95th percentiles to the median.

**Table 1:** Descriptive study of HFMD and environmental factors.

Variables	Mean ± SD	Min	25th	50th	75th	Max
Number of cases/day	130 ± 144	0	17	77	204	885
PM_2.5_ (μg/m^3^)	26.34 ± 17.23	2.33	13.73	22.55	34.86	136.52
PM_10_ (μg/m^3^)	44.1 ± 24.88	3.20	26.04	38.18	56.28	179.77
SO_2_ (μg/m^3^)	7.73 ± 3.55	3.09	5.53	6.80	8.82	56.03
NO_2_ (μg/m^3^)	32.06 ± 15.87	4.40	21.25	28.89	38.77	139.53
CO (mg/m^3^)	0.78 ± 0.26	0.39	0.57	0.71	0.90	1.92
O_3_ (μg/m^3^)	102.72 ± 45.75	19.18	66.70	94.62	128.38	316.27
Mean temperature (^。^C)	23.62 ± 5.34	3.5	19.6	24.75	28.2	33
Atmospheric pressure (hPa)	1005.63 ± 6.34	983.1	1000.8	1005.6	1010.5	1027.3
Relative humidity (%)	75.41 ± 12.68	19	70	78	84	100
Wind speed (m/s)	1.83 ± 0.77	0.3	1.3	1.7	2.2	6.1

To evaluate the potential influence of residual autocorrelation on our estimates, we performed the following two-step sensitivity analysis. First, the Pearson residuals of each main DLNM model were extracted, and residual autocorrelation was assessed diagnostically using the autocorrelation function (ACF) and the Ljung–Box test at lags of 10 and 20 days (Supplementary Table S6; Supplementary Figure S1). Second, to explicitly adjust for the first-order residual autocorrelation identified in the first step, we re-fitted each model including the first-order lagged Pearson residual as an additional covariate (hereinafter the AR(1)-adjusted model). The cumulative effect estimates of the AR(1)-adjusted models were then compared with those of the main models across all 60 exposure–percentile combinations (Supplementary Table S7). All analyses were performed using R software (version 4.4.2) with the dlnm package.

## Results

### Characterization of research data

Over the decade spanning 2013 to 2022, there were 473,695 reported HFMD cases in Shenzhen (283,519 males, 190,176 females, annual mean: 47,370 cases). Table 1 and Figure 1 summarize case counts and environmental data. Figure 2 illustrates monthly HFMD case distribution, with cases peaking from May to October (the hot season).

**Figure 1:**
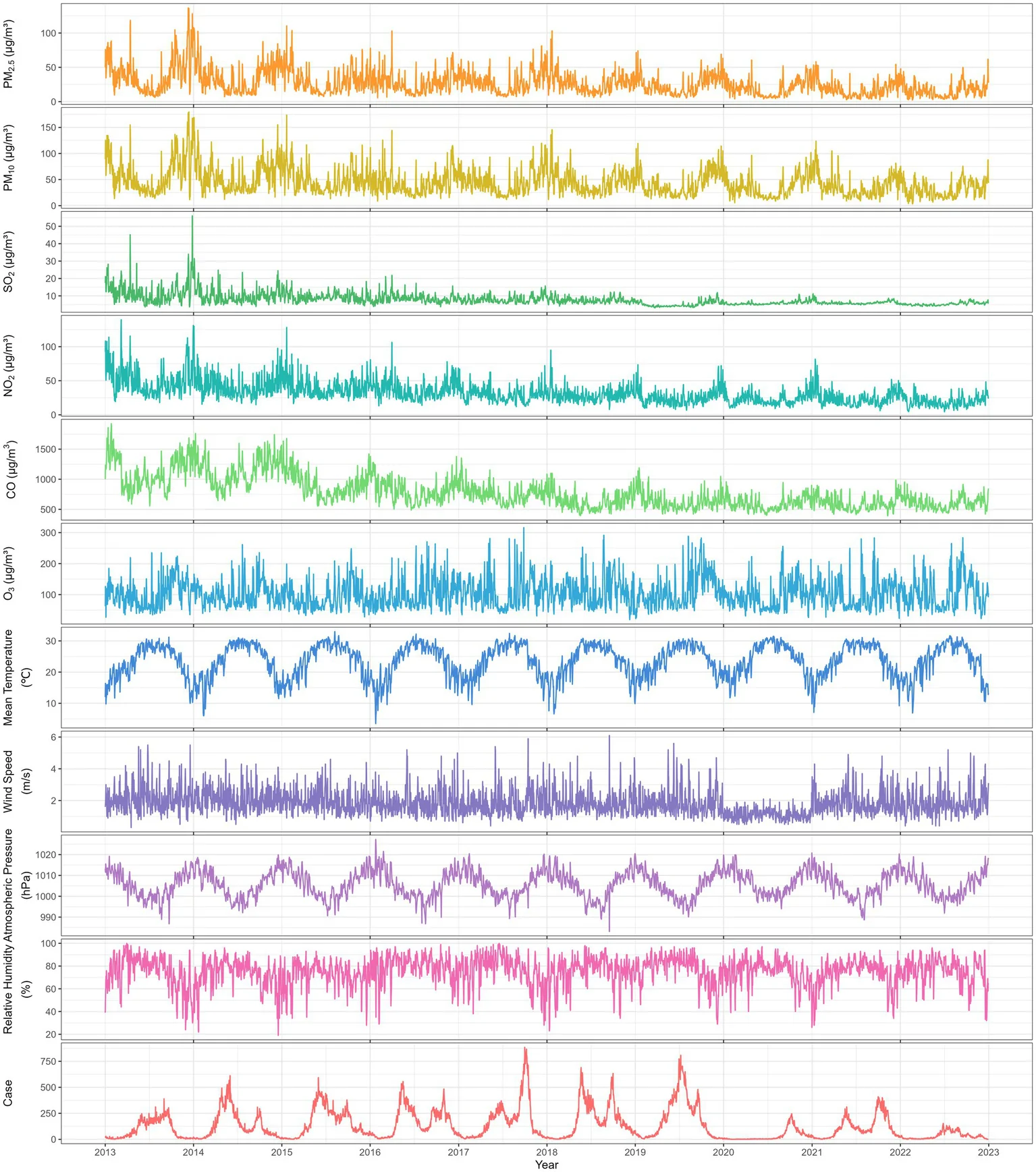
Time series of meteorological and environmental pollution factors and number of HFMD cases.

**Figure 2:**
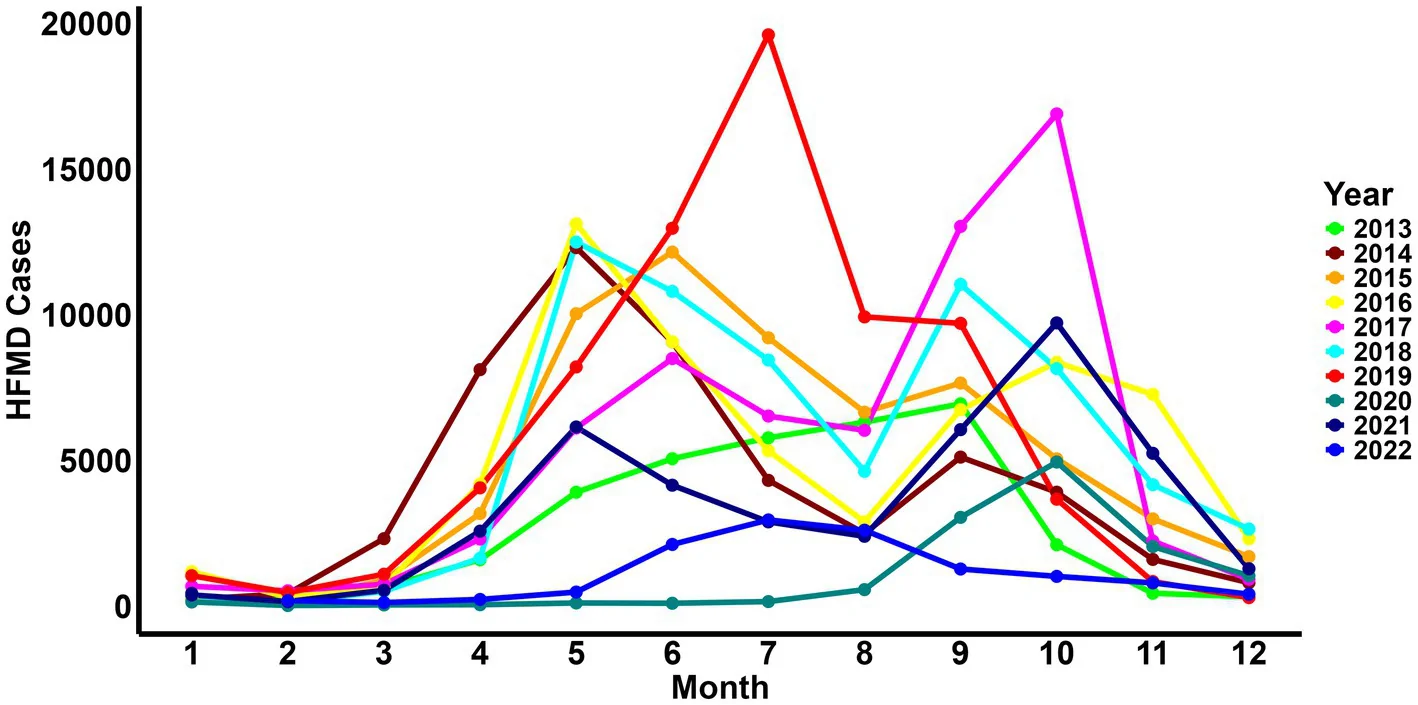
The monthly distribution of HFMD cases.

### Correlation analysis

As demonstrated by the Spearman correlation analysis (Table 2), HFMD cases were significantly positively correlated with SO₂, mean temperature, relative humidity, and wind speed, while negative correlations were identified with PM_2.5_, PM_10_, NO₂, CO, and atmospheric pressure. The strongest correlation was observed for mean temperature (*r* = 0.61), followed by atmospheric pressure which had a correlation of *r* = −0.57. In the multivariate model, PM_2.5_ and PM_10_ cannot be added to the model at the same time, which is attributable to their extremely high correlation coefficient (*r* = 0.96).

**Table 2:** Correlation analysis of environmental factors and HFMD cases.

Variables	HFMD Cases	PM_2.5_	PM_10_	SO_2_	NO_2_	CO	O_3_	Average temperature	Atmospheric pressure	Relative humidity	Wind speed
HFMD Cases	1.00***										
PM_2.5_	−0.23***	1.00***									
PM_10_	−0.20***	0.96***	1.00***								
SO_2_	0.08***	0.60***	0.60***	1.00***							
NO_2_	−0.06***	0.65***	0.63***	0.67***	1.00***						
CO	−0.10***	0.61***	0.53***	0.54***	0.702***	1.00***					
O_3_	−0.016	0.60***	0.64***	0.32***	0.23***	0.17***	1.00***				
Mean temperature	0.61***	−0.46***	−0.41***	−0.09***	−0.29***	−0.35***	−0.04*	1.00***			
Atmospheric pressure	−0.57***	0.51***	0.50***	0.17***	0.24***	0.28***	0.18***	−0.85***	1.00***		
Relative humidity	0.20***	−0.44***	−0.54***	−0.40***	−0.11***	−0.12***	−0.52***	0.23***	−0.48***	1.00***	
Wind speed	0.12***	−0.01	−0.01	0.12***	−0.08***	0.14***	−0.09***	−0.09***	0.04*	−0.15***	1.00***

*p < 0.05; ***p < 0.001.

### Distributed lag nonlinear models

We used 3-D plots and contour graphs to depict the non-linear, lagged exposure-response for air pollution and meteorological variables (Figures 3, 4). SO_2_ and CO showed associations with HFMD at high concentrations with SO_2_ showing delay effect and CO showing instant effect. At low concentrations, both NO₂ and O₃ were associated with higher HFMD risks, where NO₂ presents an immediate lag risk and O₃ has a delayed lag risk. When concentrations are high, NO₂ still retains its immediate harmful effect, while increased O₃-related risk can be detected after longer lag periods. Overall, PM_2.5_ and PM_10_ appeared to have a protective effect at high concentration levels. Mean temperature, relative humidity, and wind speed exhibited biphasic exposure–response relationships with HFMD, and the correlation was more marked for low levels of temperature and high levels of wind speed and relative humidity. Atmospheric pressure presented a significant relationship with HFMD at high exposure levels.

**Figure 3:**
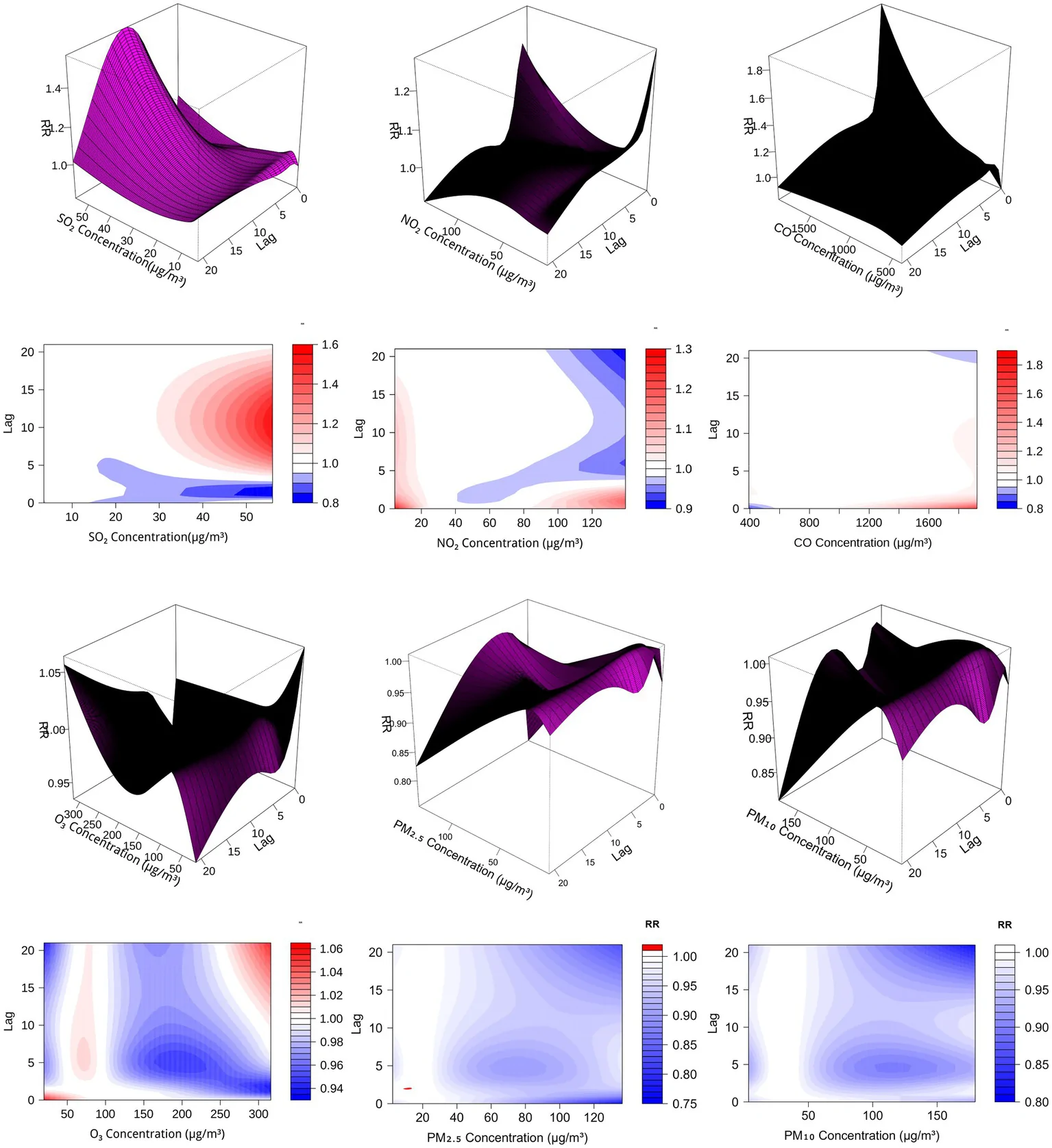
3-D plot and contour of RR along air pollution variables and lags.

**Figure 4:**
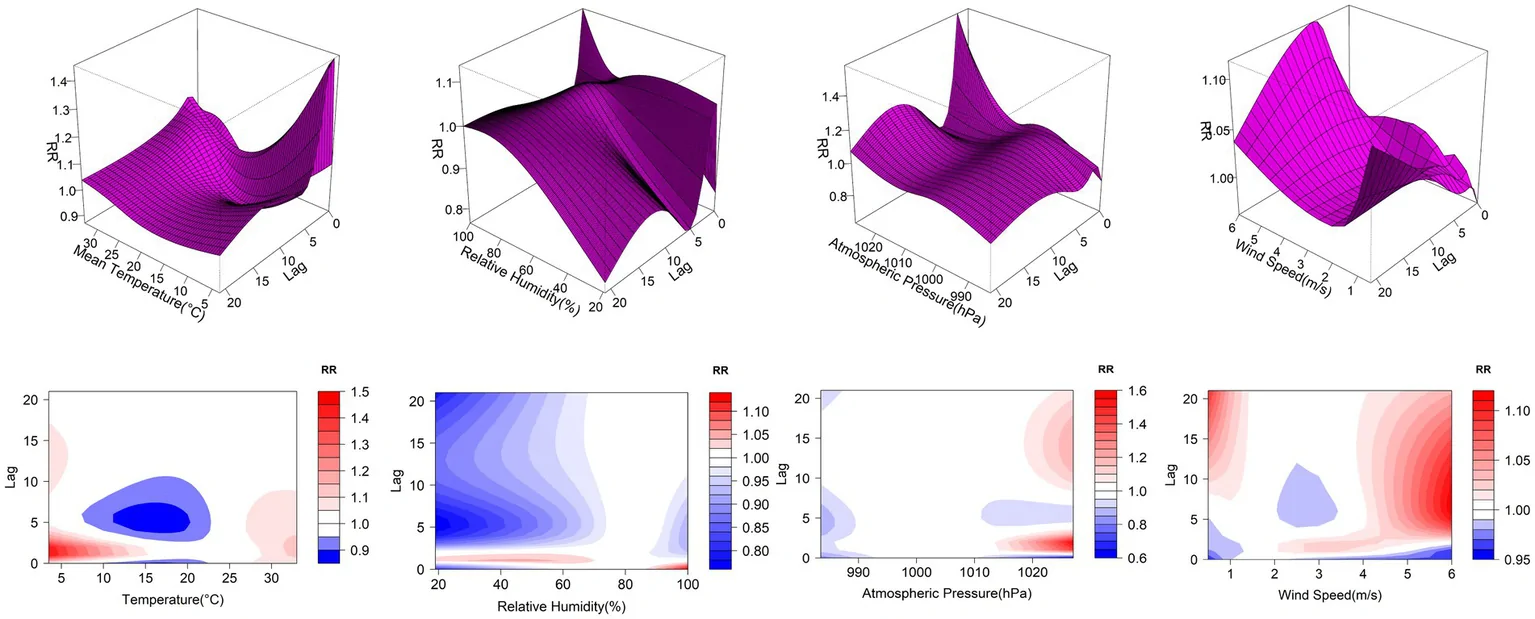
3-D plot and contour of RR along meteorological variables and lags.

Figures 5, 6 demonstrate cumulative effects: The risks of SO_2_, CO, atmospheric pressure, and wind speed increased with higher levels, though the risk of CO, atmospheric pressure, and wind speed fluctuated slightly in the process. In contrast, NO₂ showed a decreasing trend. HFMD-related risk increased with relative humidity, with high humidity corresponding to elevated incidence risk. PM_2.5_ and PM_10_ showed similar patterns, with peaks at low concentrations and protective effects at higher concentrations. Notably, O₃ and mean temperature both presented dual-risk peaks. O₃ reached risk peaks at 74.5 μg/m^3^ (RR = 1.17, 95% CI: 1.13–1.20) and 316.0 μg/m^3^ (RR = 1.15, 95% CI: 0.71–1.84); temperature had two peaks at 3.5 °C (RR = 6.67, 95% CI: 3.45–12.73) and 33.0 °C (RR = 3.43, 95% CI: 2.85–4.14).

**Figure 5:**
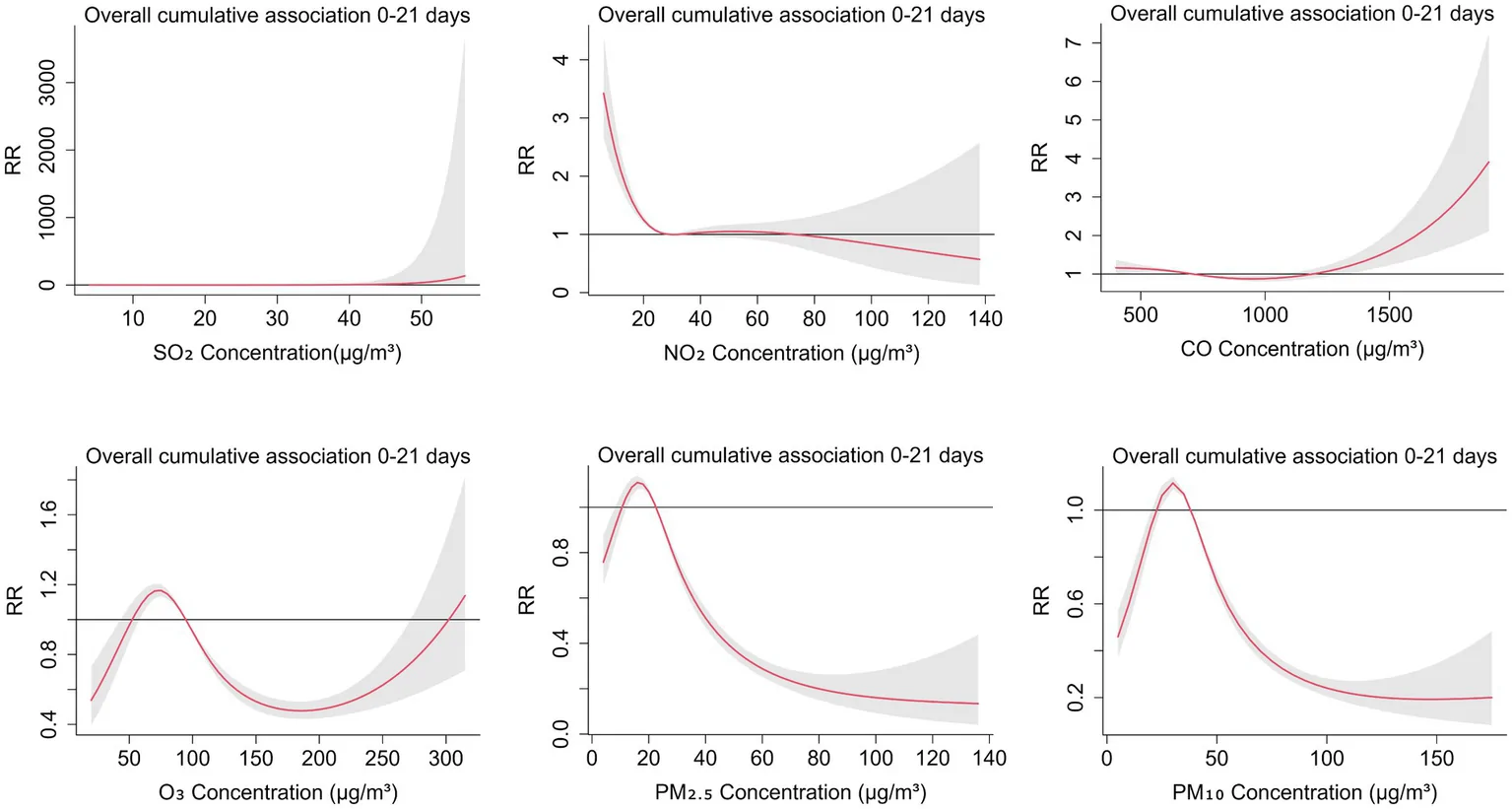
Cumulative effects of air pollution factors on HFMD incidence within 21 days.

**Figure 6:**
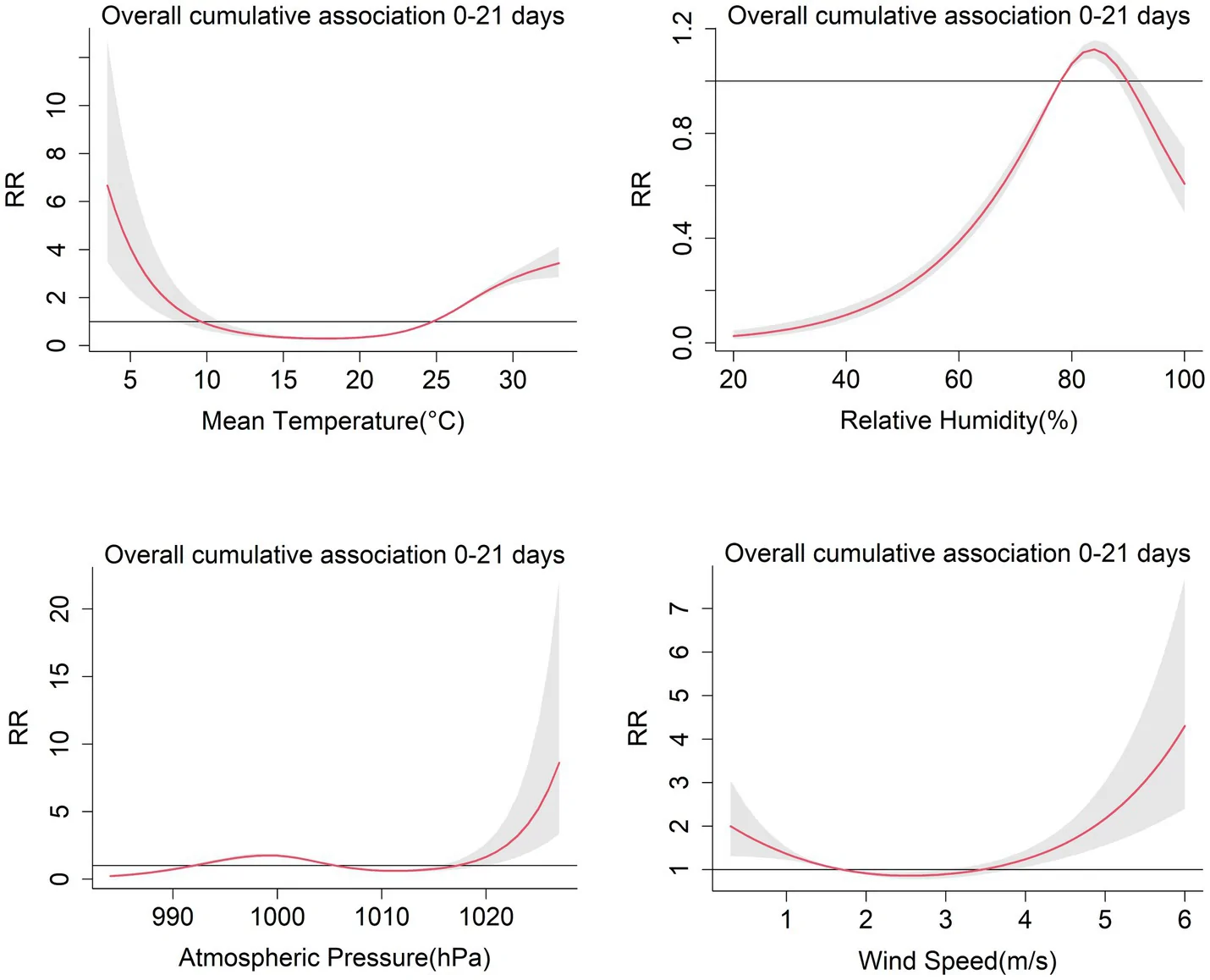
Cumulative effects of meteorological factors on HFMD incidence within 21 days.

Figure 7 reveals that extremely high levels of NO_2_ and CO, along with extremely low levels of SO₂, NO_2_, and CO showed adverse effects on HFMD. Elevated NO₂ exerted delayed-increasing harmful effects, whereas the harmful effect of high-concentration CO only occurred at lag 0–1. High-level O₃, PM₂.₅ and PM₁₀ exerted general protective effects, reaching the strongest protection during lag 4–6. Similarly, high-concentration SO₂ also showed protective effects, with its optimal-protective effect observed on lag 0. Additionally, Figure 8 demonstrates that extreme-high temperature maintained sustained harmful effects peaking within lag 0–7 days, whereas extreme-low temperature appeared to be risk at lag 1 to 2. Extremely high relative humidity caused a temporary rise in risk, while low-humidity generally produced protective effects. Exposure to high atmospheric pressure had harmful effects between lag 1 and 2, while the risk associated with low pressure rose gradually and peaked at lag 5 to 15. High wind speed caused short-term harmful effects during lag 1 to 2, whereas low wind speed presented harmful impacts that grew gradually as lag days extended.

**Figure 7:**
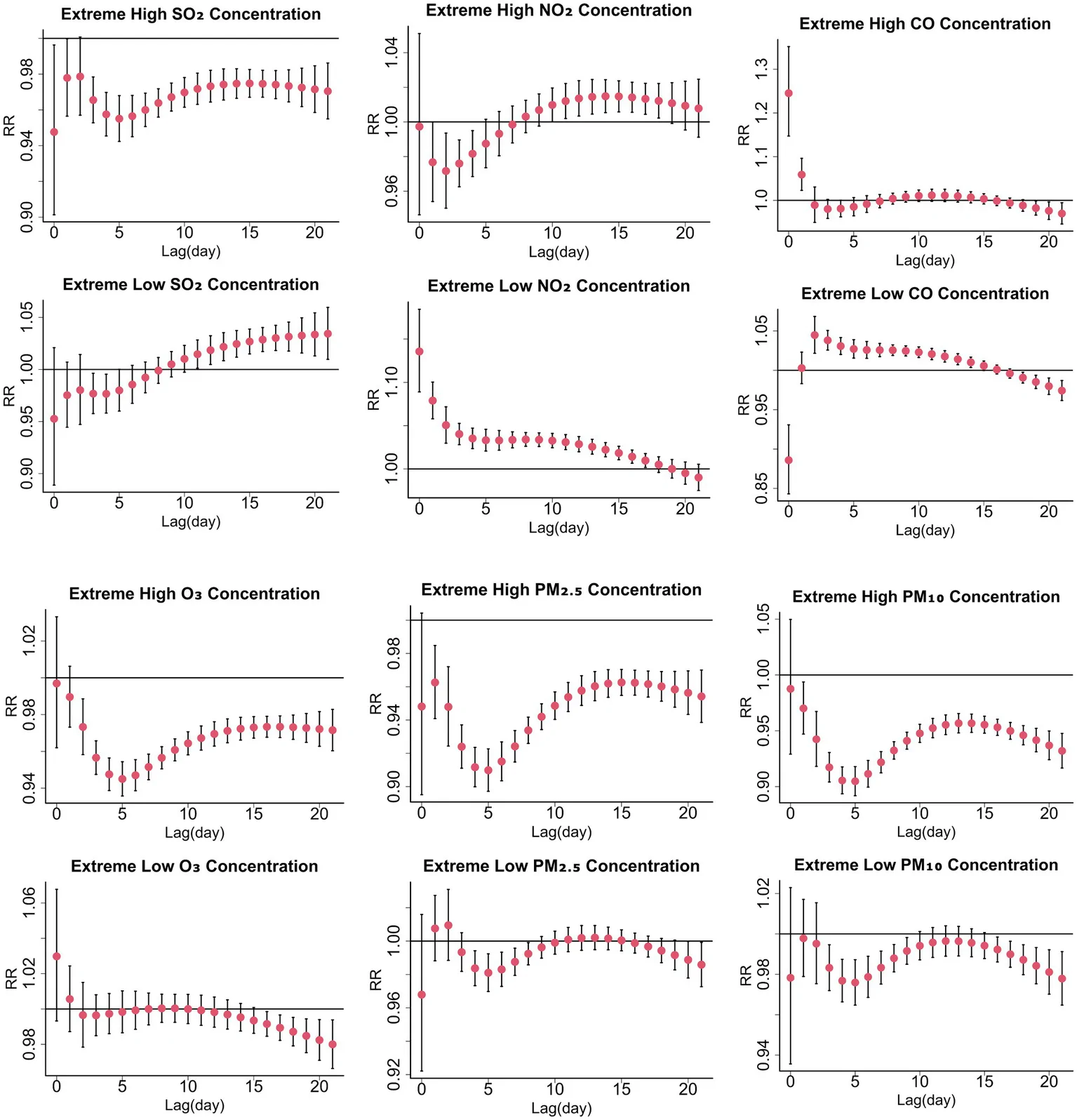
Extreme air pollutant variables with HFMD incidence.

**Figure 8:**
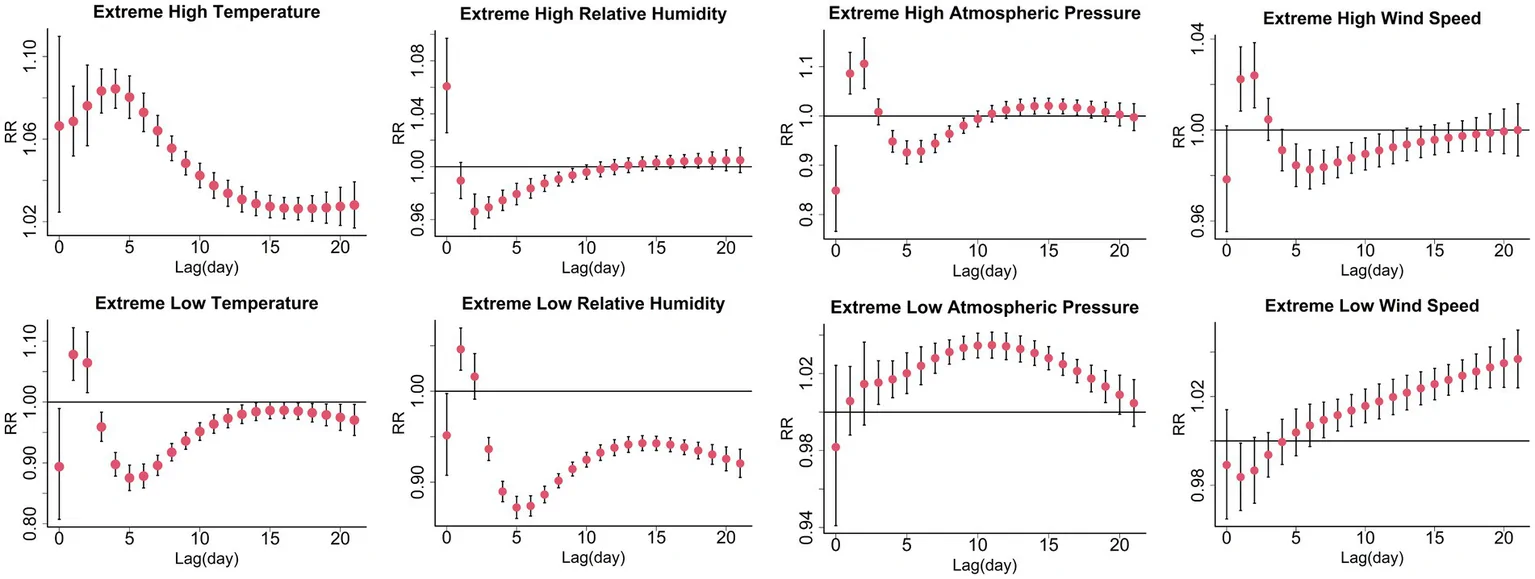
Extreme meteorological variables with HFMD incidence.

## Discussion

Climate change and urbanization have significantly transformed environmental conditions, such as air quality, temperature, and relative humidity, potentially influencing the spread of infectious diseases, including HFMD, dengue fever, Chikungunya fever, malaria, and COVID-19 (25–28). HFMD remains a key public health issue, especially among children. Understanding how air pollution and meteorological variables impact HFMD is crucial to developing targeted prevention and intervention measures. A DLNM was applied in this study to examine the relationships between air pollutants, meteorological factors, and the incidence of HFMD, accounting for week, holidays, EV71 vaccination, and COVID-19 periods.

We observed SO_2_ and CO both presented elevated cumulative risks when their concentrations were at higher levels, even though the effects observed at specific lag days were complicated. High-concentration SO_2_ showed a delayed cumulative risk, which is consistent with the results reported in earlier studies (17, 29). However, it unexpectedly displayed protective effects in single-lag analysis. This discrepancy is most likely caused by the non-linear U-shaped relationship, which is characterized differently in different time dimensions. Although the mechanism by which SO_2_ affects HFMD incidence remains unclear, it’s well-documented that SO₂ could disturb the balance of the regular intestinal microflora, which may affect the development of children’s immune systems (30). CO showed a negative Spearman correlation but increasing cumulative risk at high concentrations, with the discrepancy attributable to Spearman’s inability to capture U-shaped non-linearity. The results from Tang and Yan’s research (12, 22) also suggest that extremely high levels of CO elevate the risk of HFMD. However, the underlying mechanisms linking CO to HFMD are not well understood and warrant future investigation. NO₂ displayed a decreasing cumulative trend but U-shaped risk at extreme concentrations, with delayed-increasing harmful effects at high levels. Inconsistencies with findings in other studies (17, 31, 32) highlight the need for multi-city analyses. O_3_, a byproduct of NOx (33), exhibited different effects from NO_2_ on HFMD incidence. O_3_ presented dual cumulative peaks at 74.5 μg/m^3^ (RR = 1.17) and 316.0 μg/m^3^ (RR = 1.15, 95% CI: 0.71–1.84). High O₃ concentrations exerted protective effects after lag 1. This result is incompatible with most current studies, which found that O₃ concentration is positively correlated with HFMD incidence (12, 17, 34). However, in Yu’s study (15) elevated O₃ concentrations had a mild protective influence on HFMD. Their inference suggests that O₃ may affect infectious diseases, likely through inhibition of the persistence and reproduction of bacteria and viruses in ambient environments. PM_2.5_ and PM_10_, highly collinear (*r* = 0.96) and modeled separately, showed risk peaks at low concentrations followed by protective effects at high concentrations, consistent with inverted V-shaped curves and protective effects in Sichuan (13). During periods of high PM_2.5_ and PM_10_ concentrations, residents may modify their-related behaviors, which could reduce the risk of HFMD infection. Specifically, reduced outdoor activity time (35, 36) and increased mask-wearing on heavily polluted days (37) may partly explain this protective association.

Our study also found that mean temperature had the strongest correlation with HFMD (*r* = 0.61), and we observed a dual-peak cumulative risk pattern at both low (3.5 °C, RR = 6.67) and high (33.0 °C, RR = 3.43) temperatures. Dual peaks were also found in studies of Tianjin, Ningbo and Shijiazhuang (14, 34, 38). Elevated temperatures may exacerbate viral proliferation and boost human outdoor activities, such as summer camps,thereby facilitating virus transmission (39, 40). On colder days, human immune function might be compromised. Brief exposure to low temperatures could shorten the incubation period of microbes, leading to a higher incidence of infections (40, 41). Lagged analysis revealed asymmetric dynamics: extreme heat maintained sustained harmful effects over lag 0–21 days, whereas extreme cold produced shorter-lived risk at lag 1–2 days, suggesting that heat-related risk requires longer forecast windows. Relative humidity was positively associated with HFMD, with risk increasing monotonically — consistent with other findings (11, 22, 40, 42). Studies have demonstrated that high humidity may extend viral persistence (43). Conversely, the protective effect of low humidity aligns with accelerated viral inactivation under dry conditions, where desiccation causes structural damage to the viral capsid (44). Atmospheric pressure showed the second strongest correlation (*r* = −0.57), with cumulative risk increasing at higher pressure — consistent with Zhu et al. (42) and Sun et al. (45). The lagged analysis revealed a notably asymmetric pattern: high pressure produced short-term harmful effects, possibly reflecting behavioral changes during fair-weather conditions, while low pressure led to gradually increasing risk that persisted. The extended lag window provides significant lead time for early warning. Wind speed was positively correlated with HFMD, consistent with Guangzhou (46) and Beijing-Tianjin-Hebei (47). Our lagged analysis showed a biphasic pattern: high wind speed caused short-term harmful effects (lag 1–2) indicating that higher wind speeds may enhance airborne transmission of the virus in densely populated areas (42, 48). While low wind speed produced gradually increasing risk, possibly through facilitating the occurrence of bioaerosols (49).

Notably, the study period spanned both pre- and post-COVID-19 pandemic phases, and the temporal pattern of HFMD incidence shifted markedly after 2020. To test whether pandemic-era alterations in healthcare-seeking behavior and non-pharmaceutical interventions distorted the environmental exposure–HFMD associations, we conducted subgroup sensitivity analyses stratified by pandemic period. The effects of extreme high and low levels of meteorological factors remained broadly consistent in direction and significance across the two periods, supporting the robustness of the meteorological findings. For air pollutants, the effects of high-concentration exposure—the primary concern in air pollution health research—were likewise maintained in direction and significance across the two periods, indicating that the high-exposure findings are robust to the exclusion of the pandemic period. By contrast, several low-concentration associations (e.g., PM_2.5_, PM_10_, and O_3_) were sensitive to the exclusion of the pandemic period: when the analysis was restricted to 2013–2019, these associations changed from protective to risk-increasing in direction. This directional instability suggests that the estimated effects at the lower concentration range are not robust and may reflect, at least in part, the sparse data at low exposure levels, the relatively short post-pandemic observation period, and pandemic-related changes in exposure distributions and healthcare-seeking behavior. Accordingly, the low-concentration findings for PM_2.5_, PM_10_, and O_3_ should be interpreted with caution and regarded as exploratory. Taken together, our results suggest that the main conclusions, which are driven primarily by associations at high exposure levels, were not substantially biased by pandemic-related changes; however, the low-concentration associations identified in the full-period analysis warrant more cautious interpretation, and residual confounding by unmeasured or time-varying factors could not be fully excluded.

In the present study, the impacts of air pollutants and meteorological factors on HFMD incidence were investigated, encompassing the pre- and post-EV71 vaccination periods as well as the COVID-19 control period. Notably, our research findings offer practical guidance in two key dimensions. Firstly, our results inform public health policy by demonstrating that air pollutants and meteorological factors can affect HFMD incidence in Shenzhen, indicating the necessity of tailored interventions to address varying air quality or meteorological conditions. Furthermore, it is important to assess air quality and meteorological conditions prior to the organization of outdoor activities or public campaigns.

However, our study is not without limitations. First, this time-series ecological design is susceptible to ecological bias, and future research integrating individual-level information can achieve more in-depth and refined analysis. Second, the surveillance-based disease data may suffer from underreporting issues and incomplete case ascertainment, with unrecorded asymptomatic cases and missed mild cases further causing reporting bias, which may underestimate the actual effects of environmental factors on HFMD. Third, we adopted population-level ambient environmental monitoring data to approximate individual personal exposure, inevitably leading to exposure misclassification, which may affect the accuracy of exposure-effect association estimation. Fourth, obvious seasonal peaks of HFMD concentrated in summer and autumn were observed, and although we controlled seasonality via time splines, residual seasonal confounding could still exist. Fifth, solar radiation (sunshine duration) and precipitation were not included as model covariates; these meteorological factors may independently influence pathogen viability and children’s outdoor activity patterns, acting as potential unmeasured confounders. Sixth, this single-city study limits the generalizability of findings; multi-region comparative research is required to develop region-specific prevention strategies.

## Conclusion

This study used DLNM models to investigate non-linear and lagged associations between meteorological factors, air pollutants and HFMD incidence. Meteorological factors — particularly mean temperature and atmospheric pressure — serve as the primary environmental drivers of HFMD, exhibiting more consistent associations than air pollutants. Mean temperature showed a dual-peak distribution with persistent effects from high temperatures, while atmospheric pressure presented long-lasting impacts of low pressure. There is a positive correlation between relative humidity and the incidence of HFMD. Air pollutants showed inconsistent associations with HFMD. The seemingly protective effect of PM at high concentrations was probably not real, but rather a result of confounding by other factors. These findings support targeted interventions during extreme temperature, high humidity, and sustained low-pressure periods.

## Data Availability

The datasets presented in this article are not readily available because institutional regulations of the HFMD case data employed in this study. Requests to access the datasets should be directed to get the air pollution data and the meteorological data from the website https://opendata.sz.gov.cn/.

## Footnotes

1
https://tjj.sz.gov.cn

2
http://www.sz.gov.cn/

3
https://opendata.sz.gov.cn/
