# Supplementary material for: Ambient air pollution and meteorological factors associated with hand, foot, and mouth disease risk in Shenzhen, China: a time-series study
Source: Front Public Health. 2026 Oct 6;14:1920424. doi: 10.3389/fpubh.2026.1920424 (PMC13680450; doi:10.3389/fpubh.2026.1920424)
Supplement: Supplementary file 1 [file Supplementary_file_1.DOCX]

**Sensitivity Analyses**

To test the robustness of our primary findings, a series of sensitivity analyses were conducted by systematically varying one model parameter at a time while holding all others constant (Supplementary Tables S1–S5).

(1) Time trend df. The df for the time spline was varied at 5, 6, and 7 per year (Table S1). The lowest QAIC value was observed at df = 6 for all exposures except temperature, for which a different optimal degree of freedom was identified. Therefore, we finally selected df = 6 for the time‑stratified natural cubic spline in the main model.

(2) Maximum lag period. The maximum lag was varied at 7, 14, and 21 days(Table S2).The QAIC value reached its minimum at a maximum lag of 21 days for most exposures, with the exception of atmospheric pressure and PM₂.₅. Accordingly, a maximum lag of 21 days was selected for the primary DLNM models.

(3) Knot placement. The sensitivity of the exposure–response curves to knot location was assessed by comparing three strategies: knots at the 33^rd^ and 67^th^ percentiles, knots at the 10^th^, 75^th^, and 90^th^ percentiles, and equidistant knots. QAIC values (Table S3).QAIC values indicated that the lowest QAIC was obtained when knots were placed at the 33^rd^ and 67^th^ percentiles. Therefore, this knot‑setting scheme was adopted for the natural cubic spline of exposure variables in the main DLNM models.

(4) Collinearity diagnostics and multi‑pollutant models. Spearman rank correlation coefficients among the ten environmental factors were presented in Table 2. Variance inflation factor (VIF) was further calculated to assess multicollinearity. Initial results showed that the VIF values for both PM₂.₅ and PM₁₀ exceeded 5 when they were co‑included in the same model. After removing PM₁₀, the VIF values of all remaining covariates were less than 5 (Table S4). Consequently, PM₁₀ was excluded and only PM₂.₅ was kept in DLNM models with PM₂.₅ as the cross‑basis variable.

(5) COVID-19 period sensitivity. To assess whether the COVID-19 pandemic materially influenced the estimated associations, cumulative RRs at the 10^th^, 25^th^, 75^th^, 90^th^, 95^th^ percentile were compared between the full study period (2013–2022) and the pre-pandemic period (2013–2019) (Table S5).No substantial changes were observed for most meteorological factors and air pollutants, except that low‑concentration PM₁₀, PM₂.₅, and O₃ exhibited an obvious shift from a protective effect to a risk‑increasing effect. Minor fluctuations in relative risks were detected for several exposures.

(6) Sensitivity analysis of residual autocorrelation. Moderate-to-strong first-order autocorrelation was detected in the Pearson residuals of the main models, with ACF[1] ranging from 0.87 to 0.91 across the ten exposures (Table S6; Figure S1). The Ljung–Box test was highly significant for all exposures at both lags of 10 and 20 days (all P < 2.2 × 10⁻¹⁶; Table S6). After explicit adjustment for the first-order lagged residual, ACF[1] decreased by approximately 50–57% (to 0.38–0.43), and the lag-20 Ljung–Box statistic decreased from (27898.2–39656.1) to (8682.2–12946.6) (Table S6). The Ljung–Box test remained nominally significant after adjustment, which is expected at our sample size (n = 3,609), because the narrow 95% confidence band of the ACF (±1.96/√n ≈ ±0.033) allows even minor autocorrelation attributable to weekly and seasonal periodicity to remain detectable.

The AR(1)-adjusted estimates were largely consistent with those of the main models. Across all 60 exposure–percentile combinations, the ratio of the AR(1)-adjusted to the main cumulative RR ranged from 0.76 to 1.36, with 36 of 60 (60%) ratios within ±10% of unity (Table S7). The direction of association was identical between the two models for all 60 combinations (100%). Statistical significance (95% CI excluding unity) was preserved in 58 of 60 combinations (96.7%); in the two exceptions (NO_2_ at the 90^th^ and 95^th^ percentiles), the 95% CI crossed unity without any change in the direction of association, and in the latter case the CI of the main model already overlapped unity. Overall, these results indicate that the main findings were robust to explicit adjustment for first-order residual autocorrelation.

**Table S1. Sensitivity analysis for time trend degrees of freedom (df/year)**

| **Variable** | **Time trend degrees of freedom (df/year)** | | | **QAIC optimized** |
| --- | --- | --- | --- | --- |
|  | **df=5** | **df=6** | **df=7** |  |
| **Mean temperature** | 3665.94 | 3696.38 | 3711.67 | df=5 |
| **Relative humidity** | 3621.45 | 3531.17 | 3674.61 | df=6 |
| **Atmospheric pressure** | 3674.94 | 3657.93 | 3696.44 | df=6 |
| **Wind speed** | 3668.18 | 3606.31 | 3709.69 | df=6 |
| **SO_2_** | 3656.64 | 3588.09 | 3702.76 | df=6 |
| **NO_2_** | 3675.06 | 3622.26 | 3712.12 | df=6 |
| **PM_10_** | 3644.78 | 3604.54 | 3699.61 | df=6 |
| **CO** | 3655.99 | 3595.08 | 3695.93 | df=6 |
| **O_3_** | 3645.23 | 3573.26 | 3702.79 | df=6 |
| **PM_2.5_** | 3640.57 | 3602.31 | 3693.33 | df=6 |

**Table S2. Sensitivity analysis for maximum lag days**

| **Variable** | **Maximum lag days** | | | **QAIC optimized** |
| --- | --- | --- | --- | --- |
|  | **Lag=7** | **Lag=14** | **Lag=21** |  |
| **Mean temperature** | 3720.24 | 3711.67 | 3704.39 | Lag=21 |
| **Relative humidity** | 3705.88 | 3674.61 | 3665.03 | Lag=21 |
| **Atmospheric pressure** | 3710.4 | 3696.44 | 3699.23 | Lag=14 |
| **Wind speed** | 3717.11 | 3709.69 | 3694.15 | Lag=21 |
| **SO_2_** | 3713.04 | 3702.76 | 3697.84 | Lag=21 |
| **NO_2_** | 3713.54 | 3712.12 | 3701.66 | Lag=21 |
| **PM_10_** | 3710.27 | 3699.61 | 3694.73 | Lag=21 |
| **CO** | 3706.84 | 3695.93 | 3682.33 | Lag=21 |
| **O_3_** | 3715.35 | 3702.79 | 3711.21 | Lag=14 |
| **PM_2.5_** | 3707.24 | 3693.33 | 3684.84 | Lag=21 |

**Table S3. Sensitivity analysis for knot placement**

| **Variable** | **Knot placement** | | | **QAIC optimized** |
| --- | --- | --- | --- | --- |
|  | **33^rd^/66^th^ percentile** | **10^th^/75^th^/90^th^ percentile** | **Equidistant** |  |
| **Mean temperature** | 3957.82 | 3963.65 | 3988.09 | 33^rd^/66^th^ percentile |
| **Relative humidity** | 3661.87 | 3670.65 | 3684.49 | 33^rd^/66^th^ percentile |
| **Atmospheric pressure** | 3662.95 | 3720.84 | 3751.29 | 33^rd^/66^th^ percentile |
| **Wind speed** | 3505.36 | 3529.40 | 3530.29 | 33^rd^/66^th^ percentile |
| **SO_2_** | 3466.80 | 3498.46 | 3503.46 | 33^rd^/66^th^ percentile |
| **NO_2_** | 3575.94 | 3579.77 | 3581.96 | 33^rd^/66^th^ percentile |
| **PM_10_** | 3659.35 | 3663.33 | 3666.96 | 33^rd^/66^th^ percentile |
| **CO** | 3549.22 | 3557.79 | 3559.13 | 33^rd^/66^th^ percentile |
| **O_3_** | 3403.24 | 3414.39 | 3424.66 | 33^rd^/66^th^ percentile |
| **PM_2.5_** | 3654.35 | 3660.40 | 3667.24 | 33^rd^/66^th^ percentile |

**Table S4. Collinearity diagnostics and multi-pollutant sensitivity**

| **Variable** | **VIF (with PM₁₀)** | **VIF (without PM₁₀)** |
| --- | --- | --- |
| **PM_10_** | 18.36 | / |
| **PM_2.5_** | 17.66 | 4.19 |
| **Atmospheric pressure** | 4.42 | 4.4 |
| **Mean temperature** | 4.09 | 4.01 |
| **NO_2_** | 3.41 | 2.98 |
| **SO_2_** | 3.01 | 2.95 |
| **Relative humidity** | 2.62 | 2.17 |
| **CO** | 2.41 | 2.32 |
| **O_3_** | 1.88 | 1.85 |
| **Wind speed** | 1.29 | 1.28 |

**Abbreviations: VIF,Variance inflation factor.**

**Note: VIF > 5 indicates potentially problematic collinearity.**

.

**Table S5. COVID-19 period sensitivity analysis**

| **Variable** | **Period** | **P_10_ RR (95% CI)** | **P_25_ RR (95% CI)** | **P_75_RR (95% CI)** | **P_90_ RR (95% CI)** | **P_95_ RR (95% CI)** |
| --- | --- | --- | --- | --- | --- | --- |
| **Mean temperature** | 2013‑2019 | 0.253 (0.203, 0.316) | 0.332 (0.286, 0.386) | 1.907 (1.779, 2.044) | 2.165 (1.970, 2.380) | 2.213 (1.993, 2.456) |
|  | 2013‑2022 | 0.244 (0.202, 0.296) | 0.288 (0.254, 0.325) | 2.175 (2.052, 2.306) | 2.497 (2.306, 2.705) | 2.549 (2.334, 2.783) |
| **Relative humidity** | 2013‑2019 | 0.282 (0.254, 0.315) | 0.582 (0.544, 0.622) | 1.153 (1.113, 1.194) | 0.928 (0.881, 0.978) | 0.729 (0.674, 0.788) |
|  | 2013‑2022 | 0.357 (0.325, 0.391) | 0.648 (0.611, 0.687) | 1.117 (1.083, 1.152) | 0.936 (0.891, 0.984) | 0.770 (0.714, 0.831) |
| **Atmospheric pressure** | 2013‑2019 | 1.268 (1.131, 1.422) | 1.267 (1.154, 1.392) | 0.694 (0.630, 0.766) | 0.555 (0.449, 0.685) | 0.499 (0.367, 0.680) |
|  | 2013‑2022 | 1.258 (1.138, 1.390) | 1.342 (1.239, 1.452) | 0.665 (0.613, 0.722) | 0.589 (0.493, 0.704) | 0.583 (0.449, 0.757) |
| **Wind speed** | 2013‑2019 | 0.802 (0.701, 0.918) | 1.006 (0.956, 1.059) | 0.761 (0.710, 0.816) | 0.690 (0.614, 0.774) | 0.793 (0.697, 0.901) |
|  | 2013‑2022 | 1.019 (0.916, 1.134) | 1.147 (1.101, 1.195) | 0.726 (0.680, 0.775) | 0.629 (0.568, 0.696) | 0.677 (0.606, 0.757) |
| **SO_2_** | 2013‑2019 | 1.116 (1.000, 1.245) | 1.087 (1.026, 1.151) | 0.798 (0.763, 0.835) | 0.622 (0.561, 0.688) | 0.556 (0.485, 0.638) |
|  | 2013‑2022 | 1.102 (0.996, 1.219) | 1.117 (1.068, 1.169) | 0.723 (0.692, 0.756) | 0.527 (0.481, 0.577) | 0.458 (0.406, 0.518) |
| **NO_2_** | 2013‑2019 | 1.946 (1.721, 2.201) | 1.359 (1.289, 1.431) | 0.905 (0.842, 0.972) | 0.826 (0.724, 0.943) | 0.737 (0.626, 0.869) |
|  | 2013‑2022 | 1.945 (1.794, 2.108) | 1.341 (1.294, 1.389) | 0.857 (0.808, 0.908) | 0.769 (0.697, 0.848) | 0.746 (0.654, 0.852) |
| **PM_10_** | 2013‑2019 | 0.785 (0.705, 0.873) | 0.988 (0.943, 1.036) | 0.571 (0.528, 0.617) | 0.313 (0.278, 0.354) | 0.221 (0.191, 0.256) |
|  | 2013‑2022 | 1.220 (1.121, 1.327) | 1.198 (1.154, 1.244) | 0.653 (0.608, 0.701) | 0.437 (0.395, 0.484) | 0.342 (0.300, 0.390) |
| **CO** | 2013‑2019 | 1.606 (1.474, 1.750) | 1.349 (1.284, 1.416) | 0.784 (0.725, 0.848) | 0.854 (0.751, 0.970) | 1.091 (0.924, 1.287) |
|  | 2013‑2022 | 1.615 (1.505, 1.734) | 1.347 (1.295, 1.400) | 0.810 (0.761, 0.861) | 0.929 (0.840, 1.027) | 1.212 (1.049, 1.402) |
| **O_3_** | 2013‑2019 | 0.665 (0.594, 0.745) | 0.975 (0.927, 1.025) | 0.550 (0.508, 0.595) | 0.375 (0.337, 0.417) | 0.341 (0.306, 0.379) |
|  | 2013‑2022 | 1.223 (1.127, 1.328) | 1.225 (1.178, 1.273) | 0.679 (0.636, 0.725) | 0.508 (0.464, 0.555) | 0.442 (0.402, 0.485) |
| **PM_2.5_** | 2013‑2019 | 0.791 (0.716, 0.873) | 0.963 (0.916, 1.012) | 0.563 (0.524, 0.605) | 0.315 (0.280, 0.356) | 0.228 (0.198, 0.264) |
|  | 2013‑2022 | 1.199 (1.110, 1.296) | 1.188 (1.145, 1.232) | 0.663 (0.622, 0.708) | 0.446 (0.403, 0.494) | 0.338 (0.297, 0.385) |

**Abbreviations: RR, relative risk; CI, confidence interval.**

**Note: 2013–2022 = full study period; 2013–209 = pre-COVID-19 period (excluding 2020–2022).**

**Table S6. Ljung–Box test statistics (at lags of 10 and 20 days) and lag-1 autocorrelation (ACF[1]) of the Pearson residuals from the main and AR(1)-adjusted DLNM models for each of the 10 exposures**

| **Exposure** | **LB_10_** | | | | **LB_20_** | | | | **ACF[1]**  **Main** | **ACF[1]**  **AR(1)** |
| --- | --- | --- | --- | --- | --- | --- | --- | --- | --- | --- |
|  | **χ^2^ Main** | **P Main** | **χ^2^ AR(1)** | **P AR(1)** | **χ^2^ Main** | **P Main** | **χ^2^ AR(1)** | **P AR(1)** |  |  |
| Mean temperature | 23802.1 | **< 2.2 × 10^-16^** | 6903.1 | **< 2.2 × 10^-16^** | 39656.1 | **< 2.2 × 10^-16^** | 12006.6 | **< 2.2 × 10^-16^** | 0.90 | 0.38 |
| Relative humidity | 19346.2 | **< 2.2 × 10^-16^** | 5350.3 | **< 2.2 × 10^-16^** | 32600.7 | **< 2.2 × 10^-16^** | 9914.9 | **< 2.2 × 10^-16^** | 0.88 | 0.39 |
| Atmospheric pressure | 22829.0 | **< 2.2 × 10^-16^** | 7368.3 | **< 2.2 × 10^-16^** | 36544.3 | **< 2.2 × 10^-16^** | 12946.6 | **< 2.2 × 10^-16^** | 0.91 | 0.43 |
| Wind speed | 17390.7 | **< 2.2 × 10^-16^** | 4910.1 | **< 2.2 × 10^-16^** | 27898.2 | **< 2.2 × 10^-16^** | 9099.8 | **< 2.2 × 10^-16^** | 0.88 | 0.40 |
| SO_2_ | 17816.5 | **< 2.2 × 10^-16^** | 4967.4 | **< 2.2 × 10^-16^** | 29062.9 | **< 2.2 × 10^-16^** | 9195.0 | **< 2.2 × 10^-16^** | 0.88 | 0.40 |
| NO_2_ | 18303.8 | **< 2.2 × 10^-16^** | 4879.2 | **< 2.2 × 10^-16^** | 29886.9 | **< 2.2 × 10^-16^** | 9017.0 | **< 2.2 × 10^-16^** | 0.88 | 0.38 |
| CO | 18777.4 | **< 2.2 × 10^-16^** | 5383.1 | **< 2.2 × 10^-16^** | 30404.0 | **< 2.2 × 10^-16^** | 9941.6 | **< 2.2 × 10^-16^** | 0.88 | 0.40 |
| O_3_ | 17123.2 | **< 2.2 × 10^-16^** | 4567.3 | **< 2.2 × 10^-16^** | 28088.7 | **< 2.2 × 10^-16^** | 8682.2 | **< 2.2 × 10^-16^** | 0.87 | 0.38 |
| PM_2.5_ | 18839.5 | **< 2.2 × 10^-16^** | 5349.9 | **< 2.2 × 10^-16^** | 31390.5 | **< 2.2 × 10^-16^** | 10043.1 | **< 2.2 × 10^-16^** | 0.88 | 0.39 |
| PM_10_ | 18791.7 | **< 2.2 × 10^-16^** | 5330.5 | **< 2.2 × 10^-16^** | 31503.7 | **< 2.2 × 10^-16^** | 10025.7 | **< 2.2 × 10^-16^** | 0.87 | 0.39 |

**Abbreviations: LB, Ljung–Box test; ACF, autocorrelation function; AR, autoregressive.**

**Notes: LB_10_ and LB_20_ denote the Ljung–Box test statistic (χ²) at lags of 10 and 20 days, respectively. P values below the numerical resolution of R (< 2.2 × 10⁻¹⁶) are reported as P < 2.2 × 10⁻¹⁶. ACF1 denotes the lag-1 autocorrelation. The AR(1)-adjusted model included the first-order lagged Pearson residual of the main model as an additional covariate.**

**Table S7. Comparison of cumulative relative risks (RRs) with 95% confidence intervals (CIs) between the main and AR(1)-adjusted DLNM models at the 10^th^, 25^th^, 50^th^ (reference), 75^th,^ 90^th^, and 95^th^ percentiles of each exposure**

| **Exposure** | **Percentile** | **Value** | **RR_Main（CI_low—CI_high）** | **RR_AR1（CI_low—CI_high）** | **RR_ratio_AR1** |
| --- | --- | --- | --- | --- | --- |
| Mean temperature | 10^th^ | 16.1 | 0.091（0.077—0.108） | 0.091（0.083—0.099） | 1 |
| Mean temperature | 25^th^ | 19.7 | 0.19（0.171—0.212） | 0.178（0.168—0.189） | 0.937 |
| Mean temperature | 50^th^ | 24.8 | 1.000（1.000-1.000） | 1.000（1.000-1.000） | 1 |
| Mean temperature | 75^th^ | 28.2 | 1.77（1.665—1.881） | 1.653（1.601—1.707） | 0.934 |
| Mean temperature | 90^th^ | 29.7 | 1.608（1.451—1.781） | 1.351（1.28—1.426） | 0.84 |
| Mean temperature | 95^th^ | 30.1 | 1.524（1.354—1.716） | 1.237（1.162—1.317） | 0.812 |
| Relative humidity | 10^th^ | 58 | 0.332（0.279—0.394） | 0.384（0.348—0.424） | 1.157 |
| Relative humidity | 25^th^ | 70 | 0.793（0.715—0.881） | 0.812（0.765—0.861） | 1.024 |
| Relative humidity | 50^th^ | 78 | 1.000（1.000-1.000） | 1.000（1.000-1.000） | 1 |
| Relative humidity | 75^th^ | 84 | 0.929（0.878—0.983） | 0.946（0.917—0.977） | 1.018 |
| Relative humidity | 90^th^ | 89 | 0.735（0.666—0.811） | 0.777（0.735—0.822） | 1.057 |
| Relative humidity | 95^th^ | 92 | 0.604（0.517—0.705） | 0.658（0.602—0.718） | 1.089 |
| Atmospheric pressure | 10^th^ | 997.6 | 1.427（1.29—1.579） | 1.598（1.514—1.687） | 1.12 |
| Atmospheric pressure | 25^th^ | 1000.8 | 1.527（1.399—1.667） | 1.684（1.606—1.766） | 1.103 |
| Atmospheric pressure | 50^th^ | 1005.5 | 1.000（1.000-1.000） | 1.000（1.000-1.000） | 1 |
| Atmospheric pressure | 75^th^ | 1010.4 | 0.391（0.354—0.432） | 0.387（0.366—0.408） | 0.99 |
| Atmospheric pressure | 90^th^ | 1013.9 | 0.169（0.134—0.212） | 0.196（0.174—0.221） | 1.16 |
| Atmospheric pressure | 95^th^ | 1015.8 | 0.102（0.072—0.144） | 0.137（0.114—0.163） | 1.343 |
| Wind speed | 10^th^ | 1 | 0.903（0.773—1.054） | 0.883（0.81—0.964） | 0.978 |
| Wind speed | 25^th^ | 1.3 | 0.883（0.829—0.94） | 0.881（0.85—0.913） | 0.998 |
| Wind speed | 50^th^ | 1.7 | 1.000（1.000-1.000） | 1.000（1.000-1.000） | 1 |
| Wind speed | 75^th^ | 2.2 | 1.334（1.191—1.493） | 1.308（1.226—1.396） | 0.981 |
| Wind speed | 90^th^ | 2.8 | 1.912（1.602—2.281） | 1.823（1.649—2.014） | 0.953 |
| Wind speed | 95^th^ | 3.3 | 2.585（2.123—3.149） | 2.401（2.149—2.682） | 0.929 |
| SO_2_ | 10^th^ | 4.8 | 1.054（0.997—1.114） | 1.007（0.976—1.04） | 0.955 |
| SO_2_ | 25^th^ | 5.49 | 1.081（1.047—1.115） | 1.049（1.03—1.068） | 0.97 |
| SO_2_ | 50^th^ | 6.73 | 1.000（1.000-1.000） | 1.000（1.000-1.000） | 1 |
| SO_2_ | 75^th^ | 8.8 | 0.726（0.663—0.796） | 0.761（0.722—0.802） | 1.048 |
| SO_2_ | 90^th^ | 11.45 | 0.475（0.398—0.567） | 0.517（0.467—0.574） | 1.088 |
| SO_2_ | 95^th^ | 13.55 | 0.342（0.273—0.429） | 0.378（0.331—0.431） | 1.105 |
| NO_2_ | 10^th^ | 15.55 | 2.826（2.465—3.241） | 2.137（1.98—2.305） | 0.756 |
| NO_2_ | 25^th^ | 21.18 | 1.34（1.26—1.425） | 1.261（1.219—1.305） | 0.941 |
| NO_2_ | 50^th^ | 28.82 | 1.000（1.000-1.000） | 1.000（1.000-1.000） | 1 |
| NO_2_ | 75^th^ | 38.54 | 1.251（1.127—1.388） | 1.102（1.038—1.169） | 0.881 |
| NO_2_ | 90^th^ | 51.09 | 1.265（1.069—1.497） | 1.011（0.919—1.112） | 0.799 |
| NO_2_ | 95^th^ | 60.79 | 0.986（0.801—1.214） | 0.782（0.695—0.879） | 0.793 |
| CO | 10^th^ | 0.51 | 0.856（0.773—0.948） | 0.93（0.878—0.986） | 1.086 |
| CO | 25^th^ | 0.57 | 0.972（0.924—1.023） | 1.013（0.983—1.042） | 1.042 |
| CO | 50^th^ | 0.71 | 1.000（1.000-1.000） | 1.000（1.000-1.000） | 1 |
| CO | 75^th^ | 0.9 | 0.769（0.702—0.842） | 0.769（0.73—0.811） | 1 |
| CO | 90^th^ | 1.16 | 0.505（0.448—0.57） | 0.512（0.478—0.55） | 1.014 |
| CO | 95^th^ | 1.31 | 0.388（0.331—0.455） | 0.398（0.364—0.435） | 1.026 |
| O_3_ | 10^th^ | 52.85 | 0.691（0.595—0.803） | 0.78（0.715—0.852） | 1.129 |
| O_3_ | 25^th^ | 66.64 | 0.988（0.918—1.064） | 0.984（0.942—1.028） | 0.996 |
| O_3_ | 50^th^ | 94.57 | 1.000（1.000-1.000） | 1.000（1.000-1.000） | 1 |
| O_3_ | 75^th^ | 128.43 | 0.587（0.509—0.677） | 0.719（0.662—0.781） | 1.225 |
| O_3_ | 90^th^ | 164.91 | 0.454（0.372—0.555） | 0.617（0.55—0.693） | 1.359 |
| O_3_ | 95^th^ | 192.33 | 0.47（0.382—0.578） | 0.635（0.562—0.718） | 1.351 |
| PM_2.5_ | 10^th^ | 8.94 | 1.154（1.015—1.314） | 1.206（1.118—1.301） | 1.045 |
| PM_2.5_ | 25^th^ | 13.67 | 1.189（1.117—1.266） | 1.223（1.179—1.268） | 1.029 |
| PM_2.5_ | 50^th^ | 22.4 | 1.000（1.000-1.000） | 1.000（1.000-1.000） | 1 |
| PM_2.5_ | 75^th^ | 34.64 | 0.588（0.528—0.655） | 0.577（0.542—0.613） | 0.981 |
| PM_2.5_ | 90^th^ | 47.32 | 0.333（0.284—0.391） | 0.334（0.305—0.366） | 1.003 |
| PM_2.5_ | 95^th^ | 57.61 | 0.211（0.172—0.26） | 0.222（0.197—0.25） | 1.052 |
| PM_10_ | 10^th^ | 18.18 | 0.936（0.82—1.069） | 0.959（0.886—1.038） | 1.025 |
| PM_10_ | 25^th^ | 25.95 | 1.096（1.032—1.165） | 1.119（1.08—1.16） | 1.021 |
| PM_10_ | 50^th^ | 38 | 1.000（1.000-1.000） | 1.000（1.000-1.000） | 1 |
| PM_10_ | 75^th^ | 55.95 | 0.565（0.5—0.637） | 0.552（0.515—0.591） | 0.977 |
| PM_10_ | 90^th^ | 75.75 | 0.288（0.244—0.341） | 0.294（0.266—0.324） | 1.021 |
| PM_10_ | 95^th^ | 91.09 | 0.172（0.137—0.216） | 0.19（0.167—0.216） | 1.105 |

**Abbreviations: RR, relative risk; CI, confidence interval; AR, autoregressive.**

**Notes: Value indicates the exposure concentration at the corresponding percentile. RR_ratio_AR1 is the ratio of the AR(1)-adjusted RR to the main-model RR; values close to 1 indicate that the two estimates are similar. Cumulative RRs were calculated over the full lag period (0–21 days).**
